# Trends in congenital anomalies in Europe from 1980 to 2012

**DOI:** 10.1371/journal.pone.0194986

**Published:** 2018-04-05

**Authors:** Joan K. Morris, Anna L. Springett, Ruth Greenlees, Maria Loane, Marie-Claude Addor, Larraitz Arriola, Ingeborg Barisic, Jorieke E. H. Bergman, Melinda Csaky-Szunyogh, Carlos Dias, Elizabeth S. Draper, Ester Garne, Miriam Gatt, Babak Khoshnood, Kari Klungsoyr, Catherine Lynch, Robert McDonnell, Vera Nelen, Amanda J. Neville, Mary O'Mahony, Anna Pierini, Annette Queisser-Luft, Hanitra Randrianaivo, Judith Rankin, Anke Rissmann, Jennifer Kurinczuk, David Tucker, Christine Verellen-Dumoulin, Diana Wellesley, Helen Dolk

**Affiliations:** 1 Wolfson Institute of Preventive Medicine, Queen Mary University of London, London, United Kingdom; 2 Faculty Life & Health Sciences, University of Ulster, Newtownabbey, United Kingdom; 3 Department of Woman-Mother-Child, University Hospital Center CHUV, Lausanne, Switzerland; 4 Public Health Division of, Biodonostia Research Institute, San Sebastián, Spain; 5 Department of Medical Genetics and Reproductive Health,Children’s Hospital Zagreb, Medical School University of Zagreb, Zagreb, Croatia; 6 Department of Genetics, University Medical Center Groningen, University of Groningen, Groningen, the Netherlands; 7 National Public Health and Medical Officer Service, Hungarian Congenital Abnormality Registry, Budapest, Hungary; 8 Centro de Estudos e registo de A C, Lisbon, Portugal; 9 Department of Health Sciences, University of Leicester, Leicester, United Kingdom; 10 Paediatric department, Hospital Lillebaelt, Kolding, Denmark; 11 Directorate for Health Information and Research, Guardamangia, Malta; 12 Paris Registry of Congenital Anomalies, Inserm UMR 1153, Obstetrical, Perinatal and Pediatric Epidemiology Research Team, Center for Epidemiology and Statistics Sorbonne Paris Cité, Paris Descartes University, Paris, France; 13 Department of Global Public Health and Primary Care, University of Bergen, Bergen, Norway; 14 Division for mental and physical health, Norwegian Institute of Public Health, Bergen, Norway; 15 Department of Public Health, Health Service Executive, Kilkenny, Ireland; 16 Department of Public Health, Health Service Executive, Dublin, Ireland; 17 Provincial Institute for Hygiene, Antwerp, Belgium; 18 IMER Registry, Center for Clinical and Epidemiological Research, University of Ferrara, Ferrara, Italy; 19 Azienda Ospedaliero- Universitaria di Ferrara, Ferrara, Italy; 20 Department of Public Health, Health Service Executive, Cork, Ireland; 21 CNR Institute of Clinical Physiology, Pisa, Italy; 22 Center for child and adolescence medicine, University Medical Center of the Johannes Gutenberg University, Mainz, Germany; 23 Registre des Malformations Congenitales de la Reunion, St Pierre, Ile de la Reunion, France; 24 Institute of Health & Society, Newcastle University, Newcastle upon Tyne, United Kingdom; 25 Malformation Monitoring Centre Saxony-Anhalt, Otto-von-Guericke University Magdeburg, Magdeburg, Germany; 26 National Perinatal and Epidemiology Unit, University of Oxford, Oxford, United Kingdom; 27 Public Health Wales, Swansea, United Kingdom; 28 Center for Human Genetics,Institut de Pathologie at de Genetique, Charleroi, Belgium; 29 University of Southampton and Wessex Clinical Genetics Service, Southampton, United Kingdom; Universidad Miguel Hernandez de Elche, SPAIN

## Abstract

**Background:**

Surveillance of congenital anomalies is important to identify potential teratogens.

**Methods:**

This study analysed the prevalence of 61 congenital anomaly subgroups (excluding chromosomal) in 25 population-based EUROCAT registries (1980–2012). Live births, fetal deaths and terminations of pregnancy for fetal anomaly were analysed with multilevel random-effects Poisson regression models.

**Results:**

Seventeen anomaly subgroups had statistically significant trends from 2003–2012; 12 increasing and 5 decreasing.

**Conclusions:**

The annual increasing prevalence of severe congenital heart defects, single ventricle, atrioventricular septal defects and tetralogy of Fallot of 1.4% (95% CI: 0.7% to 2.0%), 4.6% (1.0% to 8.2%), 3.4% (1.3% to 5.5%) and 4.1% (2.4% to 5.7%) respectively may reflect increases in maternal obesity and diabetes (known risk factors). The increased prevalence of cystic adenomatous malformation of the lung [6.5% (3.5% to 9.4%)] and decreased prevalence of limb reduction defects [-2.8% (-4.2% to -1.5%)] are unexplained. For renal dysplasia and maternal infections, increasing trends may be explained by increased screening, and deceases in patent ductus arteriosus at term and increases in craniosynostosis, by improved follow up period after birth and improved diagnosis. For oesophageal atresia, duodenal atresia/stenosis and ano-rectal atresia/stenosis recent changes in prevalence appeared incidental when compared with larger long term fluctuations. For microcephaly and congenital hydronephrosis trends could not be interpreted due to discrepancies in diagnostic criteria. The trends for club foot and syndactyly disappeared once registries with disparate results were excluded. No decrease in neural tube defects was detected, despite efforts at prevention through folic acid supplementation.

## Introduction

Since thalidomide and rubella (German measles) were discovered as powerful teratogens [1,2], congenital anomaly registries have been set up to facilitate research and surveillance concerning environmental causes of congenital anomalies, and to provide early warning of new teratogenic exposures[3]. The recent increases in the prevalence of microcephaly in South America due to the infection of mothers with Zika virus during the first trimester of pregnancy, highlights the continued necessity of surveillance. [4–6] Most congenital anomalies are rare (for instance spina bifida, one of the more common anomalies, only affects one baby in every 2,000 births) and therefore it is necessary to collect information on these anomalies across a large population of births. A European network of population-based registries for the epidemiologic surveillance of congenital anomalies (EUROCAT, http://www.eurocat-network.eu/) surveys over 1.7 million births (29% of European birth population) per year from 38 high-quality multiple-source registries in 21 countries in Europe that ascertain congenital anomalies in terminations of pregnancy and births [7–9]. The EUROCAT Central Registry performs annual statistical monitoring for five and ten year pan-European trends in 25 registries [10–11]. This paper presents the latest pan-European ten year trends (2003–2012) in 61 congenital anomaly subgroups (chromosomal anomalies are excluded as recent trends in chromosomal anomalies have already been reported) [12]. Anomalies that had significant increasing or decreasing trends are investigated in greater detail, by presenting the European prevalence over the past 32 years, and the prevalence and trends within each registry over the past ten years.

## Methods

### Data

All EUROCAT registries use multiple sources of information to ascertain cases in order to cover all types of case (live birth, late fetal death (20+ weeks’ gestation), and termination of pregnancy for fetal anomaly at any gestation). Data sources, depending on registry, include maternity, neonatal, and paediatric records; fetal medicine, cytogenetic, pathology, and medical genetics records; specialist services including paediatric cardiology; and hospital discharge and child health records [9]. All cases are coded to the International Classification of Diseases (ICD) version 9 or 10 with 1-digit BPA extension. Cases can have one syndrome and up to eight malformation codes. All coding is completed using the EUROCAT guide 1.3 with minor anomalies being excluded [13].

In June 2014 anonymised aggregate data were extracted for all EUROCAT registries that had a total birth prevalence of all anomalies (including chromosomal anomalies, genetic syndromes and microdeletions) of over 2 per 100 pregnancies for the years from 2003–2012 and had data for at least nine years of the time period from 2003 to 2012. Due to ICD-9 and ICD-10 inconsistencies all registries must have used ICD10 coding for the whole ten year period from 2003. Table 1 shows the 25 registries included and their birth prevalence of all anomalies excluding chomosomal anomalies, genetic syndromes or microdeletions. Twenty one registries ascertained cases diagnosed up to at least 1 year of life and amongst these five registries had no upper age limit for registration.

**Table 1:** EUROCAT registries included in the analysis: Total number of cases (excluding chromosomal anomalies) and births surveyed from 1980 to 2012 and prevalence per 100 births.

	Years of data included		Number		
Registry	Start	Finish	All cases with an anomaly	Total births	CA Prevalence per 100 births
Hainaut	1980	2012	7,892	378,359	2.1
Odense	1980	2012	3,705	173,987	2.1
Paris	1981	2012	24,820	1,057,400	2.3
Tuscany	1980	2012	12,308	686,687	1.8
Dublin	1980	2012	13,894	743,186	1.9
N Netherlands	1981	2012	11,480	530,580	2.2
Emilia Romagna	1981	2012	14,553	911,481	1.6
Vaud	1989	2012	5,628	182,403	3.1
Zagreb	1983	2012	2,932	191,023	1.5
Malta	1986	2011	2,861	116,098	2.5
S Portugal	1990	2011	3,276	321,290	1.0
Antwerp	1990	2012	7,803	362,889	2.2
Basque Country	1990	2011	6,450	380,871	1.7
Saxony Anhalt	1987	2012	9,576	369,795	2.6
Mainz	1990	2011	2,954	72,246	4.1
Cork and Kerry	1996	2012	3,167	151,415	2.1
Wales	1998	2012	16,435	501,720	3.3
Norway	1980	2012	24,538	836,535	2.9
Isle de Reunion	2002	2012	3,567	160,551	2.2
Thames Valley	1991	2012	4,728	322,938	1.5
Wessex	1994	2012	7,110	524,372	1.4
East Midlands and South Yorkshire	1998	2012	17,066	998,655	1.7
Northern England	2000	2012	7,739	416,731	1.9
Hungary	1998	2011	36,906	1,251,751	2.9
SE Ireland	1997	2012	1,646	108,730	1.5

### Anomalies monitored

**Fig 1:**
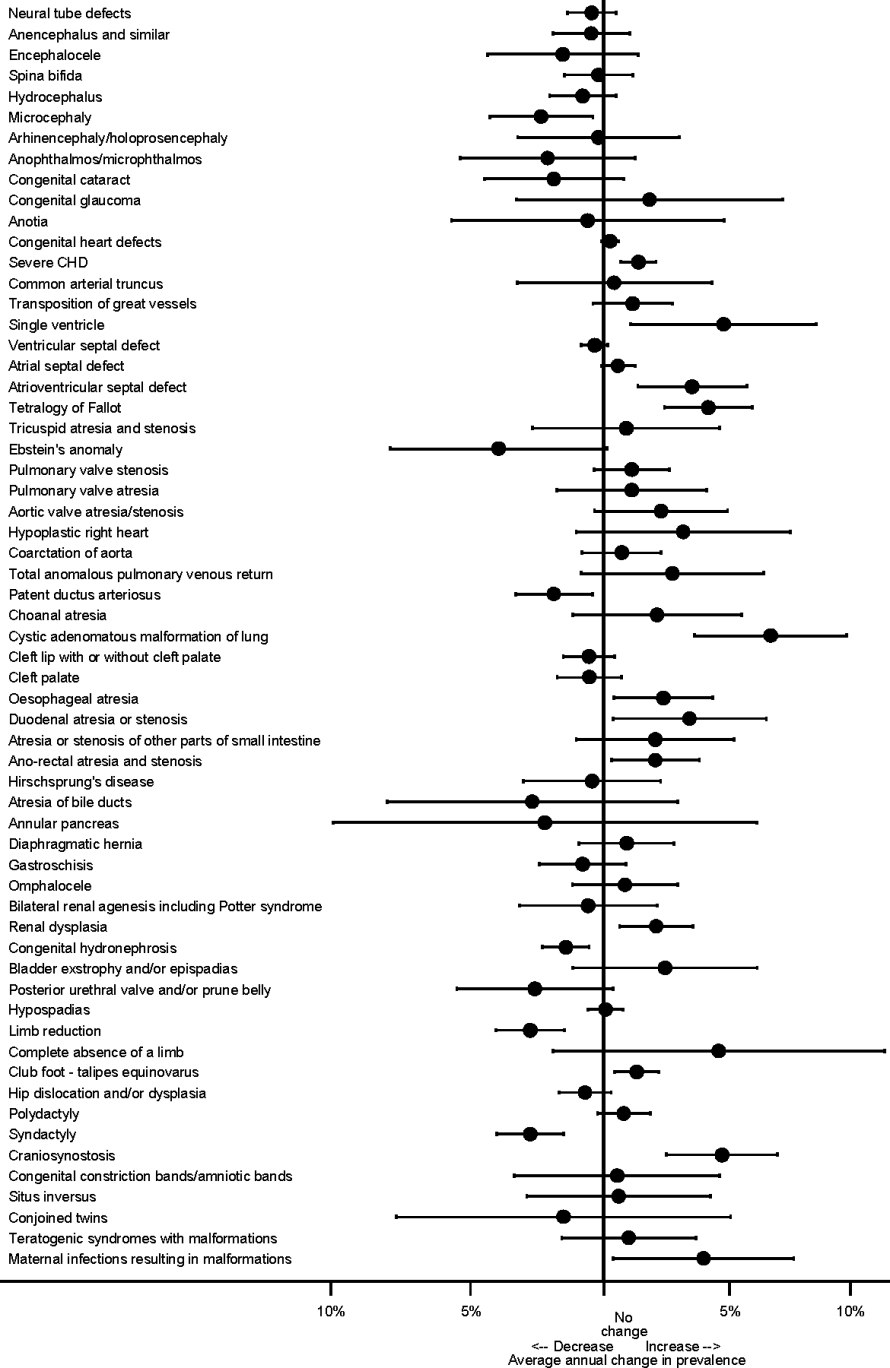
Annual change in prevalence (95% CI) from 2003 to 2012 across Europe according to anomaly group.

All the anomaly subgroups that have been monitored are given in Fig 1. All cases with chromosomal anomalies, genetic syndromes or microdeletions were excluded from the analysis (trends in the most common chromosomal anomalies have been recently reported elsewhere) [12]. Severe congenital heart defects (CHDs) have been defined as single ventricle, hypoplastic left heart, hypoplastic right heart, Ebstein anomaly, tricuspid atresia, pulmonary valve atresia, common arterial truncus, atrioventricular septal defects (AVSD), aortic valve atresia/stenosis, transposition of great vessels, tetralogy of Fallot, total anomalous pulmonary venous return, and coarctation of aorta. This was based on a previous EUROCAT study classifying CHDs into three severity groups according to perinatal mortality rate [14].

### Statistical methods for monitoring trends

The pan-European ten year trends in prevalence were examined for each congenital anomaly subgroup separately, by fitting a multi-level Poisson regression model on the number of cases of the anomaly each year within each registry, with the total number of births occurring in the area covered by the registry as the exposure. Random-effects models were used to account for potential heterogeneity in reported prevalence across registries. Results are presented as a forest plot of the average annual proportional change with its 95% Confidence Intervals (CI) in order to identify potential trends that merit more detailed investigation.

For congenital anomaly subgroups with significant increasing or decreasing trends over ten years, data over 32 years were also examined. Multilevel Poisson models were fitted for data from 1980 to 2012, with each two years entered as a categorical variable and the registries as strata. These models estimated (with 95% CI) the prevalence of each anomaly for each two year period adjusted for registry, which was necessary as some registries did not have data for the whole time period. The data were categorised into biannual categories for year of delivery, in order to reduce the sampling errors.

For congenital anomaly subgroups with significant ten year trends, the prevalence of the anomaly over the past ten years within each registry and its 95% CIs were then compared using a forest plot. The European prevalence with its 99% CIs was plotted as a vertical band, to enable visual comparison of the prevalence within each registry with the overall European estimate. The 99% level of statistical significance was chosen for the European estimate, to be consistent with the 99% CI funnel chosen below.

**Fig 2:**
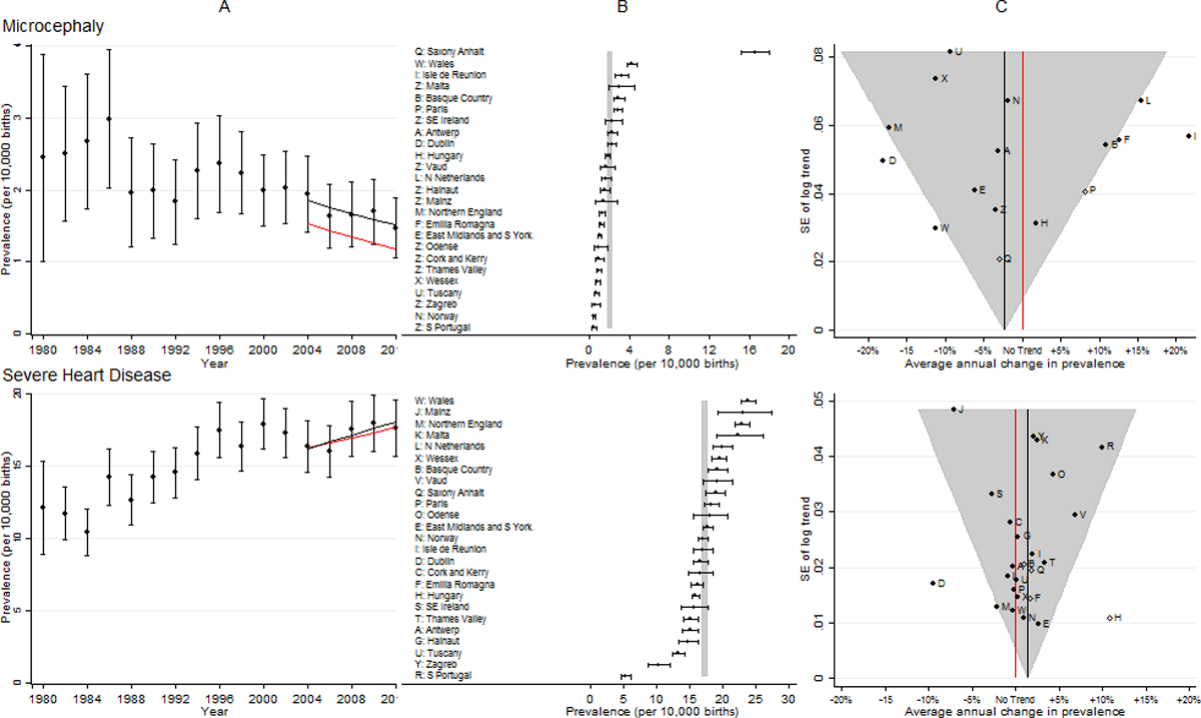
Prevalence and annual average change in prevalence for microcephaly and severe congenital heart disease. [A] European Prevalence 1981–2012 (95% CI) with trend for 2003–2012 (black line) and trend excluding outliers (red line) [B] Prevalence for 2003–2012: European (99% CI vertical grey line) and registry (95% CI) [C] Annual change in prevalence for 2003–2012: European (black line and 99% CI funnel) and registry (linear trend black dots, non-linear trend open diamonds). Red line is no trend. Severe CHD includes single ventricle, hypoplastic left heart, hypoplastic right heart, Ebstein anomaly, tricuspid atresia, pulmonary valve atresia, common arterial truncus, atrioventricular septal defects, aortic valve atresia/stenosis, transposition of great vessels, tetralogy of Fallot, total anomalous pulmonary venous return, and coarctation of aorta.

**Fig 3:**
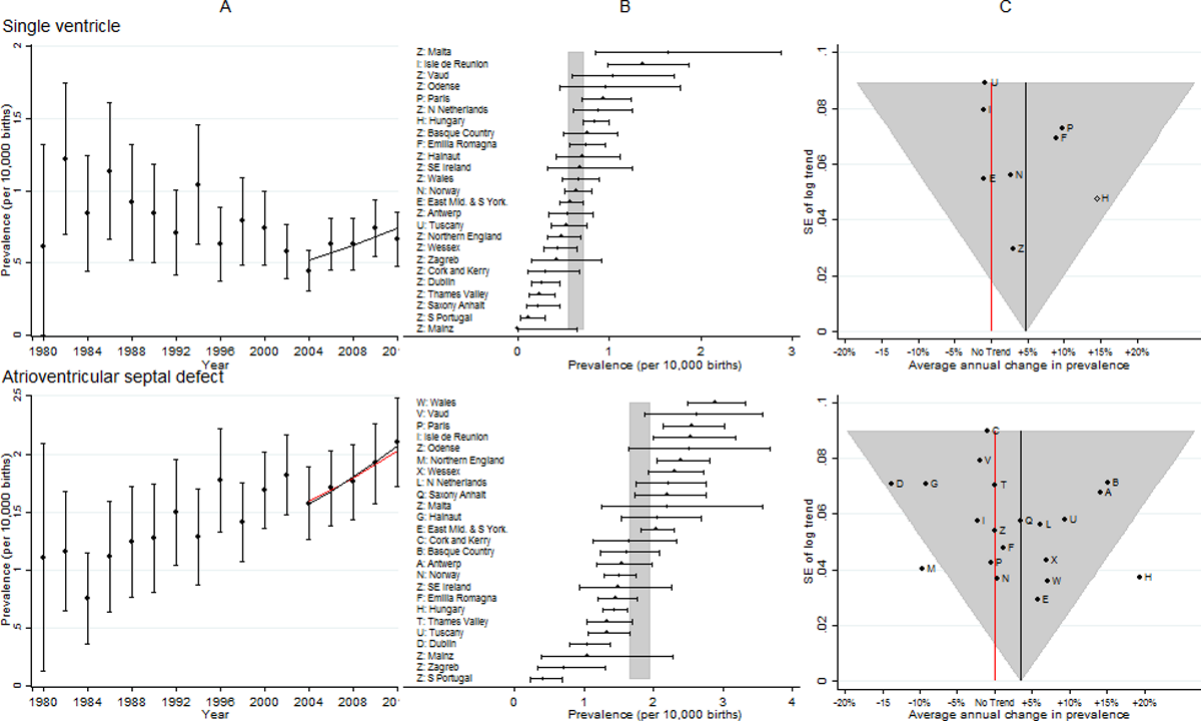
Prevalence and annual average change in prevalence for single ventricle and atrioventricular septal defect. [A] European Prevalence 1981–2012 (95% CI) with trend for 2003–2012 (black line) and trend excluding outliers (red line) [B] Prevalence for 2003–2012: European (99% CI vertical grey line) and registry (95% CI) [C] Annual change in prevalence for 2003–2012: European (black line and 99% CI funnel) and registry (linear trend black dots, non-linear trend open diamonds). Red line is no trend.

**Fig 4:**
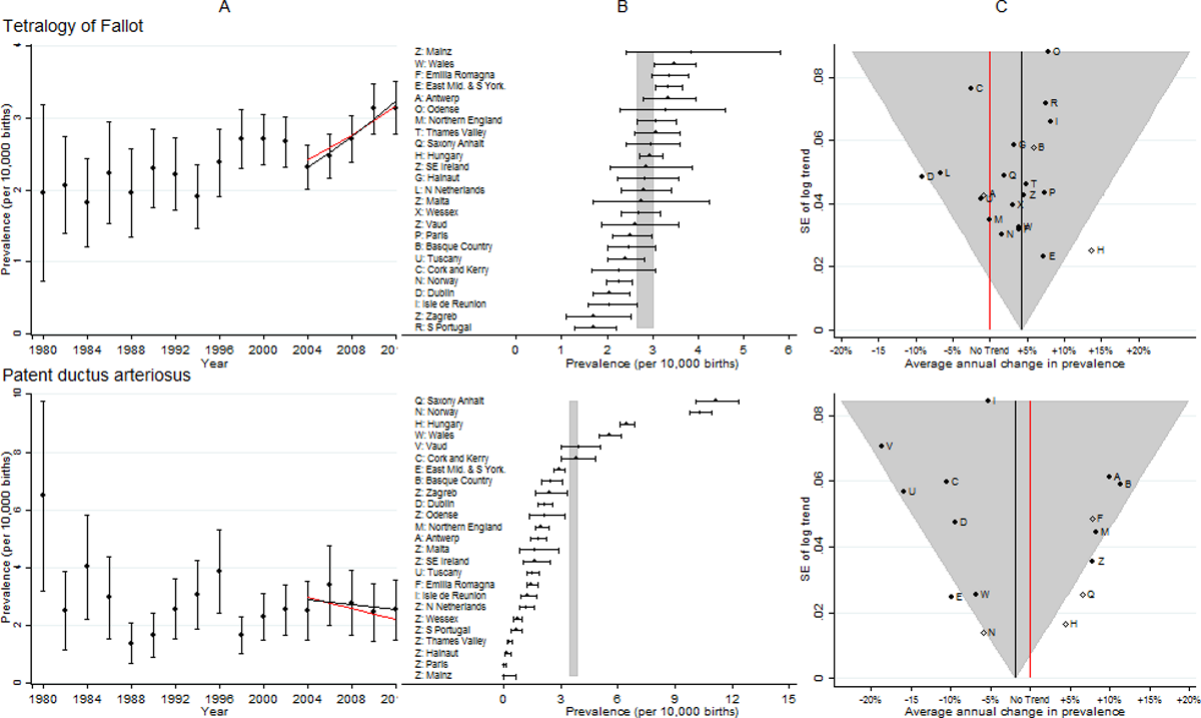
Prevalence and annual average change in prevalence for tetralogy of fallot and patent ductus arteriosus. [A] European Prevalence 1981–2012 (95% CI) with trend for 2003–2012 (black line) and trend excluding outliers (red line) [B] Prevalence for 2003–2012: European (99% CI vertical grey line) and registry (95% CI) [C] Annual change in prevalence for 2003–2012: European (black line and 99% CI funnel) and registry (linear trend black dots, non-linear trend open diamonds). Red line is no trend.

**Fig 5:**
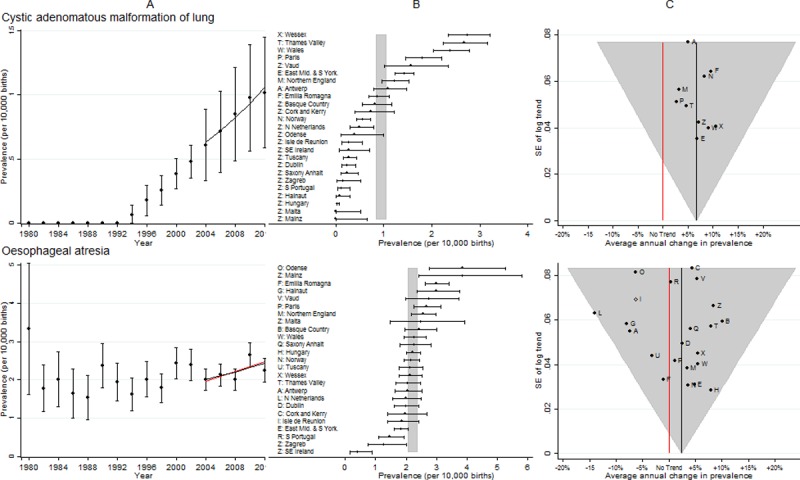
Prevalence and annual average change in prevalence for congenital cystic adenomatous malformation of lung and oesophageal atresia. [A] European Prevalence 1981–2012 (95% CI) with trend for 2003–2012 (black line) and trend excluding outliers (red line) [B] Prevalence for 2003–2012: European (99% CI vertical grey line) and registry (95% CI) [C] Annual change in prevalence for 2003–2012: European (black line and 99% CI funnel) and registry (linear trend black dots, non-linear trend open diamonds). Red line is no trend.

**Fig 6:**
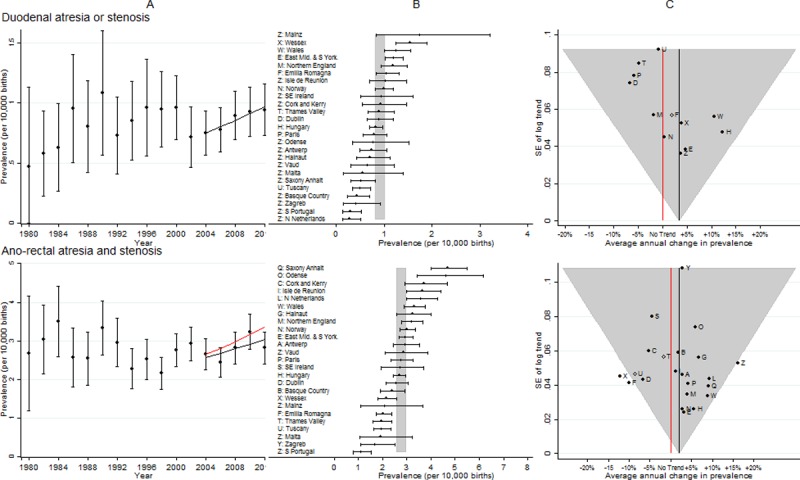
Prevalence and annual average change in prevalence for duodenal atresia or stenosis and ano-rectal atresia and stenosis. [A] European Prevalence 1981–2012 (95% CI) with trend for 2003–2012 (black line) and trend excluding outliers (red line) [B] Prevalence for 2003–2012: European (99% CI vertical grey line) and registry (95% CI) [C] Annual change in prevalence for 2003–2012: European (black line and 99% CI funnel) and registry (linear trend black dots, non-linear trend open diamonds). Red line is no trend.

**Fig 7:**
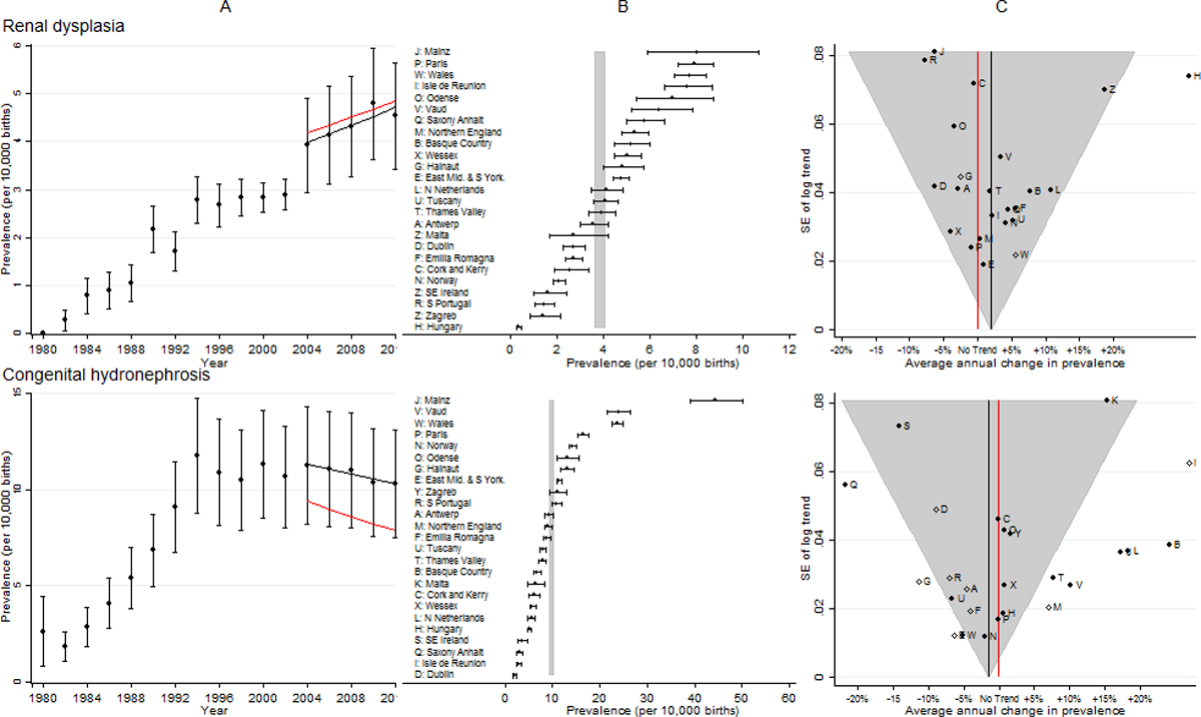
Prevalence and annual average change in prevalence for renal dysplasia and congenital hydronephrosis. [A] European Prevalence 1981–2012 (95% CI) with trend for 2003–2012 (black line) and trend excluding outliers (red line) [B] Prevalence for 2003–2012: European (99% CI vertical grey line) and registry (95% CI) [C] Annual change in prevalence for 2003–2012: European (black line and 99% CI funnel) and registry (linear trend black dots, non-linear trend open diamonds). Red line is no trend.

**Fig 8:**
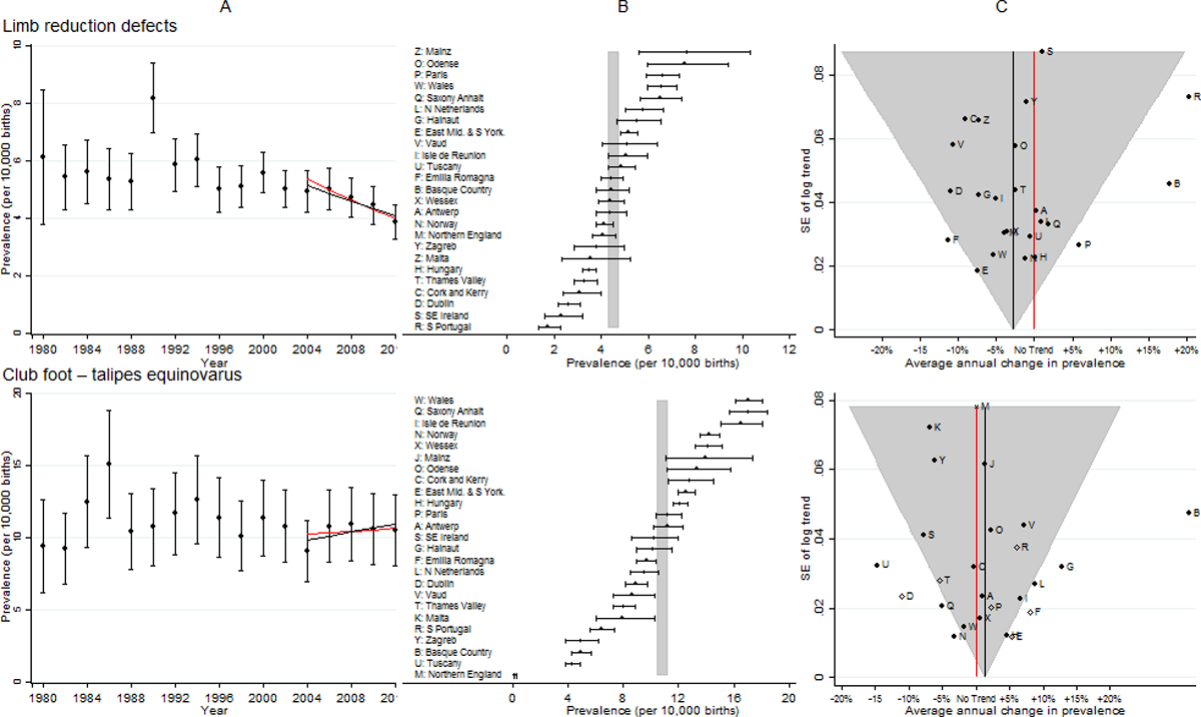
Prevalence and annual average change in prevalence for limb reduction defects and club foot–congenital talipes equinovarus. [A] European Prevalence 1981–2012 (95% CI) with trend for 2003–2012 (black line) and trend excluding outliers (red line) [B] Prevalence for 2003–2012: European (99% CI vertical grey line) and registry (95% CI) [C] Annual change in prevalence for 2003–2012: European (black line and 99% CI funnel) and registry (linear trend black dots, non-linear trend open diamonds). Red line is no trend.

**Fig 9:**
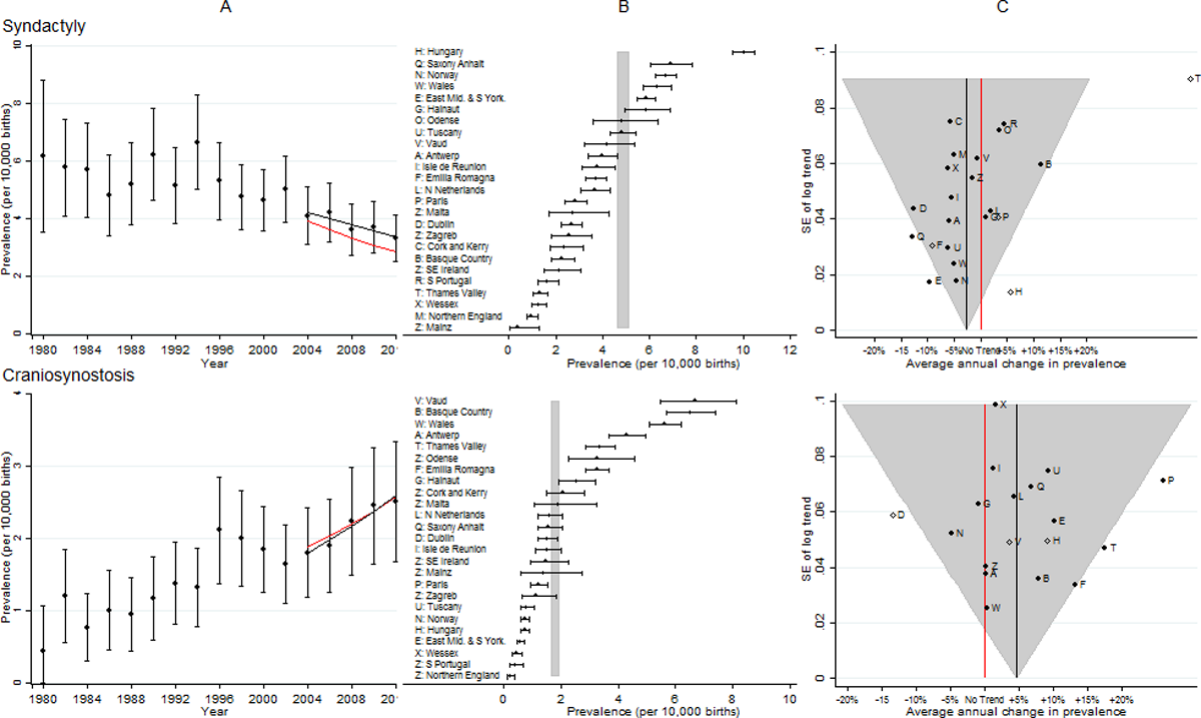
Prevalence and annual average change in prevalence for syndactyly and craniosynostosis. [A] European Prevalence 1981–2012 (95% CI) with trend for 2003–2012 (black line) and trend excluding outliers (red line) [B] Prevalence for 2003–2012: European (99% CI vertical grey line) and registry (95% CI) [C] Annual change in prevalence for 2003–2012: European (black line and 99% CI funnel) and registry (linear trend black dots, non-linear trend open diamonds). Red line is no trend.

**Fig 10:**
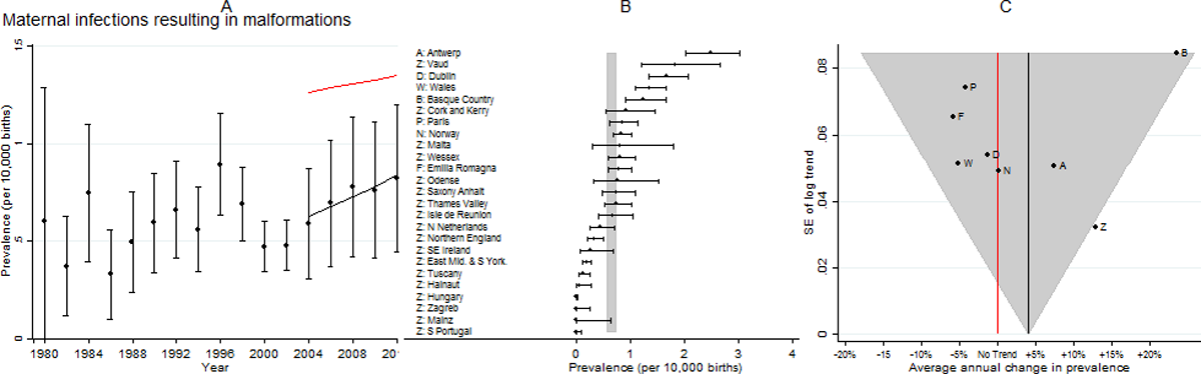
Prevalence and annual average change in prevalence for maternal infections resulting in malformations. [A] European Prevalence 1981–2012 (95% CI) with trend for 2003–2012 (black line) and trend excluding outliers (red line) [B] Prevalence for 2003–2012: European (99% CI vertical grey line) and registry (95% CI) [C] Annual change in prevalence for 2003–2012: European (black line and 99% CI funnel) and registry (linear trend black dots, non-linear trend open diamonds). Red line is no trend.

Funnel plots of the trend for each registry against the standard error of the trend were plotted for congenital anomalies with significant trends for the last 10 years. The funnel plot is symmetrical about the overall trend and visually allows for the precision of the estimates of the trends for each registry to be taken into account, with smaller registries being more uncertain about the trend, and hence higher up the axis and more likely to lie within the 99% interval, even though their estimated trends could vary from the European average. The most precise estimates are near the bottom of the graph. The two lines on the funnel plots creating the funnel are the 99% CIs of the overall trend, so any registries which lie outside these funnels are not consistent with the European trend. The 99% level of statistical significance was chosen as, with 25 registries, we would expect at least one registry to lie outside the 95% CI funnel, and only wanted to exclude from the analysis registries that were extremely likely to be outliers. The red line on the funnel plot is the line of no change in prevalence. The trend for each registry was only calculated for anomalies with at least 10 cases over a ten year period. If there were more than one registry with insufficient numbers of cases they were combined, and the trend for the combined registries was calculated and given on Figs 2–10 as “Z”. The letters in the funnel plot correspond to those in the prevalence plot, which can be used to identify the individual registries and, in particular, those included in the “Z” category.

All analyses were performed using Stata software version 12.

### Sensitivity analysis

To determine the influence of potential outliers, the analysis was repeated, excluding registries in which the prevalence over the last ten years was over five times greater than the ten year European average or whose ten year trend was outside the 99% CI funnels. “Five times greater” was chosen arbitrarily to determine outliers of the prevalence, because it was expected that the prevalence in some registries would be statistically significantly higher than the European average. The aim was to identify only those registries with coding likely to be inconsistent with the rest of Europe. The revised ten year trend was compared with the original estimated trend.

### Interpretation of trends

Before interpreting any increase or decline in total prevalence as a true increase or decline in risk, potential changes in data quality, ascertainment, screening or diagnostic methods were considered. To investigate causality, other risk factors were also considered, such as maternal age, parity, the co-occurrence of other anomalies, or whether the anomaly was isolated. The results from these investigations are considered by the EUROCAT Steering Committee, when deciding if an observed trend is likely to reflect a true change in prevalence. Full details of any additional investigations performed are given in the EUROCAT Statistical Monitoring report [15]. During this analysis, individual registries with either an unexpectedly low or high prevalence were identified. The registries in question have all been contacted and will report back to EUROCAT Central Registry, but further investigation into these discrepancies is beyond the scope of this paper. The sensitivity analyses allow the reader to determine their relative influence on the overall results.

## Results

Fig 1 presents the ten year trends according to the 61 congenital anomaly subgroups for all registries combined. There were 17 anomaly subgroups with significant trends at the 5% level of significance; 12 increasing (severe CHD, single ventricle, atrioventricular septal defects, tetralogy of Fallot, cystic adenomatous malformation of lung, oesophageal atresia, duodenal atresia/stenosis, ano-rectal atresia/stenosis, renal dysplasia, club foot, craniosynostosis and maternal infections resulting in malformations) and five decreasing (microcephaly, patent ductus arteriosus, congenital hydronephrosis, limb reduction and syndactyly). The prevalence of neural tube defects as a group, and spina bifida and anencephaly individually, have shown only a very slight non-significant reduction in the past ten years.

Table 2 gives the estimates of the European prevalence and trends (including all registries and excluding outliers) from 2003 to 2012 of the 17 anomaly subgroups identified in Fig 1. A brief summary of the interpretation of the observed trends is also given, with more details provided when each anomaly subgroup is described below. The S1 table describes each of these anomalies and details any external factors, such as changes in coding or the increased use of prenatal ultrasound scans or changes in screening, that might affect the interpretation of any trends detected.

**Table 2:** Prevalence of anomalies from 2003 to 2012, the annual proportional change during this period and the adjusted annual proportional change after excluding outliers for the 17 anomaly subgroups with statistically significant trends identified in Fig 1.

	Birth prevalence per 10,000 births (95% CI)		Annual proportional change in prevalence (95% CI)		Interpretation of results after detailed investigations within registries and consensus of EUROCAT Steering Committee (see S1 Table for further details)
Anomaly	All registries	Excluding outliers (panel B in Figs 2–10)^a^	All registries	Excluding outliers (panel C in Figs 2–10) ^a^	
1: Microcephaly	2.00 (1.89–2.11)	1.25 (1.15–1.36)	-2.4% (-4.4% to -0.4%)	-2.9% (-6.0% to 0.1%)	The diagnostic criteria must be standardised before the estimates of prevalence or trend are interpreted.
2: Severe CH^b^	17.20 (16.87–17.53)	17.08 (16.72–17.46)	1.4% (0.7% to 2.0%)	1.1% (0.3% to 1.9%)	There appears to be an increasing trend.
3: Single ventricle	0.63 (0.57–0.70)	0.63 (0.57–0.70)	4.6% (1.0% to 8.2%)	4.6% (1.0% to 8.2%)	There appears to be an increasing trend which will be monitored.
4: Atrioventricular septal defect	1.80 (1.69–1.90)	1.82 (1.70–1.94)	3.4% (1.3% to 5.5%)	3.1% (0.8% to 5.4%)	There appears to be an increasing trend.
5: Tetralogy of Fallot	2.82 (2.69–2.95)	2.83 (2.69–2.98)	4.1% (2.4% to 5.7%)	3.4% (1.5% to 5.2%)	There appears to be an increasing trend.
6: Patent ductus arteriosus	3.64 (3.49–3.79)	2.50 (2.30–2.71)	-1.9% (-3.4% to -0.4%)	-3.8% (-6.7% to -0.9%)	The slight decreasing trend is likely to have resulted from improved coding of PDA rather than a true reduction in prevalence
7: Cystic adenomatous malformation of lung	0.94 (0.87–1.02)	0.94 (0.87–1.02)	6.5% (3.5% to 9.4%)	6.5% (3.5% to 9.4%)	There appears to be an increasing trend.
8: Oesophageal atresia	2.21 (2.09–2.33)	2.22 (2.10–2.34)	2.3% (0.4% to 4.2%)	2.8% (0.8% to 4.7%)	The observed increasing trend should be interpreted with caution due to variations in prevalence since 1981.
9: Duodenal atresia or stenosis	0.91 (0.84–0.99)	0.91 (0.84–0.99)	3.3% (0.4% to 6.3%)	3.3% (0.4% to 6.3%)	The observed increasing trend should be interpreted with caution due to variations in prevalence since 1981.
10: Ano-rectal atresia and stenosis	2.77 (2.64–2.90)	2.92 (2.78–3.07)	2.0% (0.3% to 3.7%)	2.9% (1.1% to 4.7%)	The observed increasing trend should be interpreted with caution due to variations in prevalence since 1981.
11: Renal dysplasia	3.86 (3.71–4.02)	4.42 (4.25–4.60)	2.0% (0.6% to 3.5%)	1.8% (0.3% to 3.2%)	The observed increasing trend is likely to have resulted from increased uptake of prenatal ultrasounds and not a true increase in prevalence.
12: Congenital hydronephrosis	9.78 (9.54–10.03)	8.62 (8.32–8.92)	-1.5% (-2.3% to -0.6%)	-2.3% (-3.5% to -1.0%)	The diagnostic criteria must be standardised before the estimates of prevalence or trend are interpreted.
13: Limb reduction defects	4.50 (4.33–4.67)	4.49 (4.31–4.67)	-2.8% (-4.2% to -1.5%)	-3.6% (-5.1% to -2.2%)	There appears to be a decreasing trend.
14: Club foot–talipes equinovarus	10.84 (10.58–11.10)	10.36 (9.96–10.77)	1.3% (0.4% to 2.1%)	0.5% (-0.9% to 1.9%)	The increasing trend is likely to have resulted from changes in reporting rather than a true increase in prevalence.
15: Syndactyly	4.86 (4.69–5.04)	3.77 (3.59–3.96)	-2.8% (-4.1% to -1.5%)	-4.2% (-5.9% to -2.4%)	The decreasing trend is likely to have resulted from improved coding rather than a true decrease in prevalence.
16: Craniosynostosis	1.79 (1.68–1.90)	1.76 (1.65–1.87)	4.6% (2.4% to 6.7%)	4.0% (1.7% to 6.3%)	The observed increasing trend is likely to have resulted from improved follow-up by the registries rather than a true increase in prevalence.
17: Maternal infections resulting in malformations	0.65 (0.59–0.72)	1.19 (1.06–1.35)	3.9% (0.4% to 7.4%)	0.9% (-3.4% to 5.1%)	The observed increasing trend is likely to have resulted from increased screening for CMV rather than a true increase in prevalence.

^a^: Outliers are those registries whose prevalence is 5 times greater than the European prevalence or whose trends lie outside of the 99% CI (grey funnel) in panel C in Figs 2–10.

^b^: Severe CHD includes single ventricle, hypoplastic left heart, hypoplastic right heart, Ebstein anomaly, tricuspid atresia, pulmonary valve atresia, common arterial truncus, atrioventricular septal defects, aortic valve atresia/stenosis, transposition of great vessels, tetralogy of Fallot, total anomalous pulmonary venous return, and coarctation of aorta.

### Microcephaly

Fig 2 panel B shows the prevalence in Saxony Anhalt was more than five times the European prevalence (due to a more lenient definition of the reduction in brain size judged to be microcephalic), and therefore the data from Saxony Anhalt were subsequently excluded from the trend analysis. The two registries that had the most inconsistent trends compared with the European trend were Isle de la Reunion (increasing more than expected) and Dublin (decreasing more than expected). Excluding Saxony Anhalt (due to its high prevalence) and the five registries that were outside the 99% grey funnel from the trend analysis (panel C) caused the estimated trend to have a slightly greater decrease, and the overall prevalence to be significantly lower (panel A). This data on the prevalence of microcephaly in EUROCAT has been published demonstrating that any changes in the prevalence of microcephaly due to the Zika virus would be unlikely to be detected in most of Europe [16] due to the expected low prevalence of the Zika virus in most of Europe plus the discrepant prevalence in the registries shown in Fig 1.

### Severe CHD

Fig 2 shows that South Portugal and Zagreb had unexpectedly low prevalence of these anomalies (panel B). Hungary had an unexpectedly high increase in prevalence (panel C), which could be explained by its very low prevalence at the start of the ten year period. Due to data protection and logistical issues, the ascertainment of cases of severe CHD in Dublin was known to have decreased after 2009, which would explain the decreasing trend observed in this register (panel C). Removal of Dublin and Hungary had a negligible effect on the European increasing trend estimate (panel A).

### Single ventricle

Fig 3 shows the European prevalence is less than 1 per 10,000 births, which means that only seven registries had sufficient cases to analyse the ten year trend individually. The cases from the remaining registries were combined (Z symbol on Fig 3 panel C). The prevalence and increasing trend was consistent amongst all registries.

### Atrioventricular septal defects (AVSD)

Fig 3 panel B shows the prevalence of AVSD was consistent across registries. As with severe CHD, Hungary had an unexpectedly high increase in prevalence, which could be explained by its very low prevalence at the start of the ten year period. There was a decreasing trend in Northern England that was inconsistent with the European trend. Due to data protection and logistical issues, the ascertainment of cases of AVSD in Dublin was known to have decreased after 2009, which would explain the decreasing trend observed in this register. Removal of Hungary and Northern England had a negligible effect on the European increasing trend estimate.

### Tetralogy of fallot (TOF)

Fig 4 shows the results were similar to those for severe CHD, Hungary again had a large increasing trend and Dublin a decreasing trend that were not consistent with the European trend. Removal of Dublin and Hungary had a negligible effect on the European increasing trend estimate.

### Patent ductus arteriosus (PDA)

Fig 4 shows the high prevalence in Saxony Anhalt, which is being investigated. In Norway, the prevalence began to fall from 2007, and by 2011/12 it was 8 per 10,000, high but not exceptionally so. Hungary again had a large increasing trend. After removal of the five outliers (Hungary, Norway, East Midlands, Saxony Anhalt and the combined ten registries with small numbers of cases (Z symbol panels B & C) a decreasing European trend remained.

### Congenital cystic adenomatous malformation of lung (CCAM)

Fig 5 shows of the seven registries with the highest prevalence, five were from the UK. Only nine registries had enough cases to enable trends in the prevalence across the ten years to be examined. Data from the remaining registries were combined (point Z on the funnel plot panel C). There was a consistent 6.5% increase in overall prevalence per annum. When examined separately isolated CCAM appeared to be increasing, however, CCAM in combination with another major congenital anomaly in a different organ system did not (data not shown).

### Oesophageal atresia

Fig 5 shows the prevalence was reasonably consistent across all EUROCAT registries. The northern Netherlands was the only registry with an unexpectedly large decreasing trend. After removal of the northern Netherlands, an increasing European trend remained.

### Duodenal atresia or stenosis

Fig 6 shows the prevalence was so low that 13 registries had too few cases to analyse the trends individually. There was an increasing trend in duodenal atresia or stenosis, with both the prevalence and the trend being consistent amongst all registries.

### Ano-rectal atresia and stenosis

Fig 6 shows both Wessex and Emilia Romagna had decreasing trends that were inconsistent with the overall trend, and after removing them from the analysis, the European increasing trend became greater.

### Renal dysplasia

Fig 7 shows the prevalence was reasonably consistent across all EUROCAT registries; only Hungary was an outlier, with an extremely low prevalence at the start of the ten year period, and hence an extremely large increase over the ten year period. After removing Hungary from the analysis, the European increasing trend remained.

### Congenital hydronephrosis (CH)

Fig 7 shows there was considerable variation in CH prevalence across Europe, with Mainz, Vaud and Wales being particularly high. There was a large number of registries that were not consistent with the overall European trend, and when they were excluded, the prevalence differed considerably from the original estimate, with the European decreasing trend remaining.

### Limb reduction defects

Fig 8 shows the prevalence was reasonably consistent across Europe. From 2004 there was a decline in prevalence, although three registries (South Portugal, Basque country and Paris) appeared to have had increasing trends that were significantly different from the overall trend, and Emilia Romagna had a significantly greater decline than the overall decline.

### Club foot–congenital talipes equinovarus (CTEV)

Fig 8 shows that there were several registries with inconsistent trends when compared with the European average. Northern England only reported club foot if other anomalies were present, isolated cases were not reported, hence the prevalence was low. When the data from the registries with inconsistent trends was removed, there was no longer an increasing trend in club foot.

### Syndactyly

Fig 9 shows there was a sudden decrease in prevalence and then a continued decrease. However the prevalence varied considerably between registries and there were several registries with inconsistent trends when compared with the European average. Thames Valley was a clear outlier, with a very strong increasing trend. This was due to increased diagnosis as a result of raising awareness about syndactyly, due to a research project on Apert’s syndrome in the area covered by the registry (syndactyly occurs in babies with Apert’s syndrome).

### Craniosynostosis (CS)

Fig 9 shows there was a large disparity in prevalence between registries and three registries had inconsistent trends compared with the average for Europe. After removal of the outliers the increasing European trend remained.

### Maternal infections resulting in malformations

Fig 10 shows the prevalence varied considerably between registries, with Antwerp having the highest prevalence. Due to the extremely low prevalence, a large number of registries did not have sufficient cases for the trend to be estimated individually, but when these registries were combined (to form the Z group) the trend was increasing and inconsistent with the European trend. Excluding these registries resulted in a considerably higher overall prevalence, because all the registries with a low prevalence were excluded, and an increasing trend remained. The fetuses with maternal infections resulting in anomalies in EUROCAT were from mothers infected with mainly cytomegalovirus, almost no rubella and a few toxoplasmosis.

## Discussion

This study analysed the pan-European trends in prevalence in 25 different registries for 61 different congenital anomaly subgroups. We concluded that for five congenital anomaly subgroups (severe CHD, single ventricle, AVSD, tetralogy of Fallot and cystic adenomatous malformation of the lung) the observed increase in prevalence from 2003 to 2012 was likely to reflect a true increase in prevalence, and that for limb reduction defects the observed decrease in prevalence was likely to reflect a true decrease in prevalence. Three congenital anomaly subgroups (oesophageal atresia, duodenal atresia/stenosis and ano-rectal atresia/stenosis) showed around an annual 3% increase in prevalence. However, the ten year trends for these three anomalies have been interpreted cautiously, as there have been very large fluctuations in prevalence over the past 30 years for all three, and the current prevalence is similar to that in the 1990’s. The study also observed trends in prevalence which were judged to be due to increased screening (renal dysplasia and maternal infections), improved follow up and hence more accurate diagnosis (PDA and craniosynostosis), changes in reporting (club foot), or changes in coding (syndactyly). There were two anomaly subgroups for which the results were not interpreted due to definitional differences between registries (microcephaly and congenital hydronephrosis). It is important to establish routinely the difficulties in the surveillance of microcephaly in order to know how to interpret any future increases in the prevalence, due to new infections such as the Zika virus or other teratogenic exposures, or due to changes in case finding for microcephaly.

Severe CHD as a whole and single ventricle, AVSD and tetralogy of Fallot individually all showed increases in prevalence. The European trend has been reported in an earlier paper [17] using EUROCAT data to 2007. The model used in that paper estimated the trend in four year and three year groups, for example, 2001 to2003 and 2004 to 2007. In this study we presented data in two year groups. Hence the fall seen in our data from 2001/2 to 2003/4 and 2005/6 corresponds to the decrease from 2004 to 2007 in the previous study. Additional data available up to 2012 shows that after a decrease from 2001/2 to 2003/4 there appeared to be an increasing trend. The main risk factors identified for CHD are diabetes, increased body mass index (BMI), smoking, alcohol consumption and the use of assisted reproductive technologies (ART) [18–25]. The increases in diabetes, BMI and ART use in Europe may therefore explain some of the observed increased prevalence [26]. Folic acid has been linked to reducing the risk of CHD, but the evidence is inconclusive [23]. However, the prevalence of neural tube defects (NTDs) (which are strongly associated with folic acid consumption) has not decreased and, therefore, it is unlikely that any changes in folic acid consumption would have influenced the trends in CHD.

The prevalence of cystic adenomatous malformation of the lung has increased in Europe, partly due to the increased availability and quality of prenatal ultrasounds. However the prevalence and the increasing trends in prevalence were particularly high in England and Wales. Since 2004 over 96% of pregnant women in the UK have been offered fetal anomaly scans at 18–22 weeks. The increasing trend may reflect an improvement in the quality of the scans. Since 2007, in Northern Netherlands a 20 week prenatal ultrasound scan has been offered to all pregnant women. However, cases with cystic adenomatous malformation of the lung are only included if they are confirmed postnatally. There are no known risk factors for this anomaly, but further investigation will be required if the increasing trend continues.

The prevalence plot of limb reduction defects over time (Fig 8 panel A) shows a clear outlier in 1989/90. This is due to several EUROCAT registries having a higher prevalence in these two years. Several studies at the time reported an association between limb reduction and early chorion villus sampling (CVS) [27,28]and it was quickly recommended that CVS should not be performed before 70 days gestation. It is likely that performing early CVS was responsible for this temporary increase. There are no clear reasons for the observed decreases.

The prevalence of NTDs as a group and spina bifida and anencephaly individually showed only a very slight non-significant reduction in the past ten years. The trends have been analysed in greater detail by Khoshnood et al [29]. Taking a folic acid supplement before conception and during the first trimester has been shown to reduce the prevalence of NTDs, and if this health advice was being adopted by the majority of women we would have expected to see reductions in the prevalences of these anomalies [30]. Mandatory fortification in many countries across the world has been shown to reduce the prevalence by up to 40% [31–34]. Such fortification should be urgently considered throughout Europe.

This study has a number of strengths, the main one being its size. Data were available on 250,000 congenital anomalies from 11.5 million births across Europe over 30 years. Many congenital anomalies are extremely rare, and therefore it is essential to combine data from many registries to be able to determine whether the prevalence is changing. For example, in 16 registries there were too few cases of cystic adenomatous malformation of lung to derive a trend, but when combined there is a very clear increasing trend. A further strength of the study was the use of the EUROCAT Steering Committee in the interpretation of the data. It is essential when analysing data from a large variety of sources covering a large range of anomalies that the results are interpreted by a group of expert clinicians and epidemiologists, with detailed knowledge of the individual registries over a long period of time. As a result of the Steering Committee’s expert opinion and external information, out of the 17 statistically significant trends observed, only six were considered to reflect true changes in prevalence.

The EUROCAT data analysed here covers 600,000 births per year, capturing around 10% of all births in Europe. Caution should therefore be exercised in interpreting these results as representative of Europe. A weakness of the study is that all the analyses depended on consistent coding across all the registries. Guides and coding tips are regularly produced to help ensure that this occurs [13]. However, for some anomalies, such as microcephaly, the large variation in prevalence according to individual registries indicates that there remain differences in diagnosis of and discrepancies in coding practices for these congenital anomalies. Examining the data in detail, as in this study, highlights these discrepancies and enables further coding advice to be given. A further weakness of the analysis is that each anomaly was considered in isolation. This is potentially unrealistic as anomalies from the same organ system may be likely to have similar trends. For example, the patterns in prevalence of oesophageal atresia, duodenal atresia or stenosis and ano-rectal atresia and stenosis all appear similar, and this should perhaps be considered when analysing any of them individually.

This is the most comprehensive summary of congenital anomaly prevalence in Europe. We have identified that there are still some congenital anomalies that are continuing to increase in Europe, in particular severe CHDs. Risks factors for these anomalies include diabetes and obesity, both of which are known to be increasing in Europe. Public health prevention policies should therefore continue to focus on these two conditions. The issue of increasing obesity in pregnancy is being addressed in the USA with the *Healthy People 2020* including objective number 16.5 to increase the proportion of women entering pregnancy with a normal weight from 52.5% in 2007 to 57.8% by 2020.[35] The expected decrease in NTDs did not occur, and therefore the issue of adequate folic acid consumption needs to be addressed. The lack of observed reductions in prevalence in any of the congenital anomaly subgroups (apart from limb reduction defects) indicates the importance of adopting the EUROCAT (European Surveillance of Congenital Anomalies)/EUROPLAN Recommendations on policies to be considered for the primary prevention of congenital anomalies in the European National Plans and Strategies on Rare Diseases [36]

## Supporting information

S1 Table Description of the congenital anomalies with statistically significant ten year trends. (PDF)
