# Supplementary material for: Trends in congenital anomalies in Europe from 1980 to 2012
Source: PLoS One. 2018 Apr 5;13(4):e0194986. doi: 10.1371/journal.pone.0194986 (PMC5886482; doi:10.1371/journal.pone.0194986)
Supplement: S1 Table — (PDF) [file pone.0194986.s001.pdf]

S1 Table : Description of the congenital anomalies with statistically significant ten year trends

Congenital Anomaly	Description
1: Microcephaly	Microcephaly is defined as “a reduction in the size of the brain with a skull circumference more than three standard deviations below the mean for sex, age and ethnic origin” [1]. However, a previous study of microcephaly registered by EUROCAT registries found that clinicians had different values of skull circumference at which microcephaly would be diagnosed [2]. The prevalence in Saxony Anhalt was more than 5 times the European prevalence, due to a more lenient definition of the reduction in brain size judged to be microcephalic.
2: Severe CHD	Severe CHDs have been defined as single ventricle, hypoplastic left heart, hypoplastic right heart, Ebstein anomaly, tricuspid atresia, pulmonary valve atresia, common arterial truncus, atrioventricular septal defects (AVSD), aortic valve atresia/stenosis, transposition of great vessels, tetralogy of Fallot, total anomalous pulmonary venous return, and coarctation of aorta, based on a previous EUROCAT study classifying CHDs into three severity groups [3], which were determined according to the perinatal mortality rate for each of these groups.
3: Single ventricle	Single ventricle is a severe congenital cardiac anomaly where the heart comprises only one complete ventricle with an inlet valve and an outlet portion even though the outlet valve is atretic.
4: Atrioventricular septal defect (AVSD)	AVSD is a central defect of the cardiac septa and the common atrioventricular valve.
5: Tetralogy of Fallot (TOF)	TOF is a congenital heart defect which involves at least three and often four anatomical abnormalities of the heart. It is the most common cyanotic heart defect.
6: Patent ductus arteriosus (PDA)	PDA is a congenital anomaly where a neonate's ductus arteriosus fails to close after birth. PDA should only be recorded as a congenital anomaly in EUROCAT when it is the only CHD in term infants (≥37 weeks gestation) [1]. There is some uncertainty as to whether all registries excluded PDA if there was an unknown gestation. In addition, if a baby was born at term with pulmonary hypertension or another critical illness like a pulmonary infection, then an echocardiogram often shows PDA in the first week but this resolves itself. This is

	particularly a problem if the registry uses hospital discharge codes without a clinical evaluation. Any decreasing trend is likely to reflect improved diagnosis.
7: Cystic adenomatous malformation of lung (CCAM)	CCAM is a rare lung lesion easily diagnosed on a prenatal ultrasound scan. The prevalence is likely to reflect the availability of high quality prenatal ultrasound scans. Some registries only include CCAM cases that were confirmed postnatally which would result in a lower prevalence.
8: Oesophageal atresia	Oesophageal atresia is the most common anomaly of the oesophagus and is characterised by a complete discontinuity of the oesophagus, with or without an abnormal connection between the oesophagus and the trachea; oesophageal stenosis is rare.
9 : Duodenal atresia or stenosis	Duodenal atresia or stenosis is the absence or complete closure of a portion of the lumen of the duodenum.
10: Ano-rectal atresia and stenosis	Ano-rectal atresia and stenosis is the absence, closure, or constriction of the rectum or anus.
11: Renal dysplasia	Renal dysplasias are a diverse group of congenital anomalies characterised as malformations of the kidney tissue. Advances in prenatal ultrasound have led to greater diagnosis of renal anomalies in general and therefore the prevalence is likely to reflect the availability of high quality prenatal ultrasound scans [4].
12: Congenital hydronephrosis (CH)	CH is an obstruction of the urinary flow from kidney to bladder. The EUROCAT definition of CH was revised in 2003 to include only those cases "if renal pelvis is 10 mm or more after birth'. An earlier EUROCAT study noted the extremely wide variation in prevalence across Europe and concluded that some of the variation maybe due to the availability of prenatal ultrasound, which is the main indication for diagnosis [5].
13: Limb reduction defects	Limb reduction defects occur when a part of or the entire arm or leg of a fetus fails to form completely during pregnancy; the limb may be reduced from its normal size, or some parts or an entire limb may be missing.
14: Club foot – congenital talipes equinovarus (CTEV)	Club foot is a complex deformity of the foot also known as CTEV, and is caused by the abnormal development of a fetuses bones, ligaments and muscles. It must be distinguished from postural club foot, which is caused by the position of the fetus in the womb putting pressure on the foot, causing it to be moulded in an abnormal position. On presentation, the foot position will be flexible and easy to correct. In some registries it may not be possible to accurately distinguish between postural and non-postural club foot and hence these registries refrain from reporting uncertain cases.

15: Syndactyly	Syndactyly is a condition where two or more digits of the hands or the feet of a fetus are fused together. In 2004 EUROCAT changed the coding to exclude cases of 2-3 syndactyly of the feet that were judged to be minor anomalies. Any decrease in prevalence may reflect the increasing use of the correct coding.
16: Craniosynostosis (CS)	CS is a condition where the cranial sutures, which are open at birth to allow the newborn's head to mould as it passes through the birth canal and to facilitate rapid growth in infancy and childhood, fuse prematurely. About half of all cases are not diagnosed until one month after birth, with this proportion increasing over the past ten years. Improvements in follow – up may therefore lead to an increased prevalence of craniosynostosis.
17: Maternal infections resulting in malformations	Maternal infections, (including Toxoplasmosis, Rubella and cytomegaloviruses) are known to be associated with a collection of congenital anomalies including heart defects and eye and ear anomalies [6]. The fetuses with maternal infections resulting in malformations in EUROCAT are from mothers infected with mainly CMV, almost no rubella and a few toxoplasmosis. If there is routine screening for CMV and if a woman has a baby with an anomaly her CMV status will be known, and if positive the baby is likely to be categorised into the maternal infections group. In areas without such screening babies will only be categorised into the maternal infections group if they have subsequently been tested for CMV, which often will not occur.

Supplementary References

1. EUROCAT. EUROCAT Guide 1.3: Instruction for the Registration of Congenital Anomalies. 2005. http://www.eurocat-network.eu/content/EUROCAT-Guide-1.3.pdf. Accessed 23rd March 2015.
2. Dolk H, De Wals P, Hamilton F. Investigation of an Apparent High Rate of Microcephaly in Glasgow and Implications for Epidemiologic Surveillance. In: EUROCAT Report 1", Brussels, Belgium. Department of Epidemiology, Catholic University of Louvain. 1986
3. Dolk H, Loane M, Garne E; European Surveillance of Congenital Anomalies (EUROCAT) Working Group. Congenital heart defects in Europe: prevalence and perinatal mortality, 2000 to 2005. Circulation 2011;123: 841-849.
4. Winding L, Loane M, Wellesley D, Addor MC, Arriola L, Bakker MK, Bianchi F, Calzolari E, Gatt M, Haeusler M, Lelong N, Mullaney C, Scarano G, Tucker D, Wiesel A, Garne E. Prenatal diagnosis and epidemiology of multicystic kidney dysplasia in Europe. Prenat Diagn 2014;34:1093–1098.
5. Garne E, Loane M, Wellesley D, Barisic I; EUROCAT Working Group. Congenital hydronephrosis: prenatal diagnosis and epidemiology in Europe. J Pediatr Urol 2009;5:47-52.
6. Rasmussen SA, Erickson JD, Reef SE, Ross DS. Teratology: from science to birth defects prevention. Birth Defects ResA Clin Mol Teratol 2009;85:82-92.
